# Metabolic effects occurring in irradiated and non-irradiated red blood cellular components for clinical transfusion practice: An in vitro comparison

**DOI:** 10.4102/ajlm.v7i1.606

**Published:** 2018-12-06

**Authors:** Faieqa Adams, Gregory R.M. Bellairs, Arthur R. Bird, Oluwafemi O. Oguntibeju

**Affiliations:** 1 Western Province Blood Transfusion Service, Cape Town, South Africa; 2 Department of Biomedical Sciences, Cape Peninsula University of Technology, Cape Town, South Africa

## Abstract

**Background:**

Storage lesions occur in red blood cell products when potassium ions, haemoglobin and lactate dehydrogenase are released into the extracellular plasma due to post-irradiation storage or cellular degeneration. The South African blood transfusion establishments do not comply with the universal leucocyte-reduction policy due to cost and the current HIV pandemic. Various studies regarding storage lesions have been completed in well-developed countries but not in Cape Town, South Africa.

**Objective:**

This study aimed to determine cellular degeneration occurring in non-irradiated and irradiated red blood cells (RBC) by comparing the measured biochemical and haematological indices during storage of up to 42 days.

**Method:**

Eighty whole blood units were collected from voluntary, non-remunerated donors. Blood components tested weekly until expiry were whole blood, RBC concentrate, leucocyte-reduced RBC concentrate (pre-storage) and paediatric RBC concentrate (*n* = 20). Ten units per product were irradiated and 10 were not. Evaluations included potassium, sodium, glucose, lactate dehydrogenase, phosphate, haemoglobin, haematocrit, mean corpuscular haemoglobin, mean corpuscular haemoglobin concentrate, mean cell volume and plasma haemoglobin. Plasma haemolysis levels were calculated using an approved formula.

**Results:**

The haemolysis levels evaluated on Day 35 and Day 42 were higher than the recommended 0.8%, whereas results for the non-irradiated components up to expiry were all below 0.8%.

**Conclusion:**

This study confirms that gamma irradiation aggravates the RBC storage lesions. The products tested yielded similar results to other studies in developed countries, however the South Africa transfusion medicine policy should remain unchanged.

## Introduction

The administration of red blood cell (RBC) products enhances intravascular oxygen-carrying capacity and is considered a vital treatment for patients with anaemia triggered by haemorrhage due to surgery, trauma or various haemoglobinopathies or malignancy.^1^ Most clinicians agree that RBC products deteriorate as soon as they leave the donor, despite an ongoing debate about increasing the storage period of blood products while still ensuring the safety and efficacy of stored blood.^2^ Storage lesions include, but are not limited to, acidosis due to lowering of pH, decreased 2,3-diphosphoglyceric acid concentrations, low adenosine triphosphate levels, a decrease in intracellular potassium and increase in cytoplasmic sodium concentrations, lipid peroxidation and oxidative stress to Band 3 structures and partial membrane loss via vesicle formation.^3^

Damage to the cell membrane causes haemolysis and potassium leakage, although these increases are not regarded as being clinically significant except in the following situations: when patients present with renal failure, at the onset of hyperkalaemia or when aged whole blood is transfused to neonates. When dealing with ill neonates, the potassium levels on storage may become significant, especially when large volumes are transfused.

Haemoglobin, lactate dehydrogenase (LDH) and potassium cations are released into the supernatant due to oxidative damage caused by storage lesions.^4^ Formation of haemoglobin microparticles due to the loss of RBC membrane structural integrity are caused by glucose consumption. The membrane becomes deformed due to an increase in osmotic fragility and spheroechinocytes are formed. While some storage lesions may occur within days or weeks, elevated potassium and LDH levels may be detected within hours of storage.^3^ All these changes affect blood rheology and may add to transfusion difficulties.^5^

### Whole blood and component therapy

Whole blood (WB) is collected from the donor, and is the primary material used for component processing with an expiry period of 35 days when stored at 1 °C to 6 °C. WB is normally administered for neonatal exchange transfusions to treat hyperbilirubinaemia as a result of haemolytic disease of the newborn. This treatment decreases the neonatal bilirubin level by removing the Rh-D-positive RBCs and the circulating maternal allo-anti-D.^6^ In adults, it is also clinically indicated to rectify massive haemorrhage due to trauma, surgical or obstetrical causes.^7^

Component therapy is based on the transfusion of a specific component as this allows more patients to benefit from a single WB donation, while reducing the need for entire WB transfusions.^8^ The RBC concentrate (RBCC) is the component indicated for patients suffering from anaemia, obstetric haemorrhage, surgery or patients with an acute blood loss of more than 30% total blood volume to improve oxygen delivery to body tissues.^9^

RBC products can be obtained by processing a WB donation by either using a centrifugation or apheresis method.^10,11,12^ In South Africa, the standard procedure used is the centrifugation method. The plasma and buffy coat are extracted and distributed between the sterile, interconnected but separate satellite bags. Saline-adenine-glucose-mannitol is then added to the packed red cells which allows the RBCC to be stored for 42 days. The buffy coat-poor method produces a better quality plasma yield due to the high centrifugal forces used, and leucocyte levels in RBCC are reduced without filtration.^12^ Another advantage is the reduction of micro-aggregate formation during storage.^10^ This is the standard product prepared for the majority of RBC transfusions in South Africa and all RBC products are stored at 1 °C to 6 °C.^6^

A normal RBCC unit may contain about 1 to 2 billion leucocytes, which may cause various transfusion-associated complications.^13^ Leucocytes are known to increase cellular damage and cause adverse transfusion reactions in recipients, such as allo-immunisation to human leucocyte antigens, non-haemolytic febrile transfusion reactions, transfusion-associated graft versus host disease and transfusion-associated lung injury. Leucocytes may also be a pathogenic haven for certain transfusion-associated leukotropic viruses such as Epstein-Barr virus and cytomegalovirus. B-lymphocytes may be regarded as vectors for the variant Creutzfeldt-Jakob disease prions.^14^ Transfusion-related immunomodulatory effects, which include post-operative infections leading to mortality due to multi-organ failure, is another transfusion reaction.^6,15^

In order to minimise or prevent these adverse reactions, blood transfusion establishments prepare leucocyte-reduced and irradiated blood component products. Blood is filtered soon after collection as fragmenting granulocytes may cause cellular damage, immune complications or transmit leukotropic viruses from donor to patient.^12,15^ The RBCC unit may be filtered by physically inserting the spike of the filter into the RBCC unit via a port on the bag, but this reduces the expiry time to 24 h post-filtration. Another method is to add the filter to the RBCC using a sterile connecting device to maintain sterility. The latter procedure is used to produce a pre-storage leucocyte-reduced RBCC, with a 42-day expiry when stored at 1 °C to 6 °C.

Unlike many well-developed countries, South Africa does not abide by the universal leucocyte-reduced policy, as the pre-storage leucocyte-reduced RBCC component is produced only when needed. This is due to the high cost involved and the HIV pandemic. There is also no evidence to suggest that patients transfused with filtered RBCC will evade the transmission of Creutzfeldt-Jakob disease.^16^

The filtered adult RBCC unit may be further modified by equally dividing the adult unit to produce two paediatric units of approximately 130 mL each or four infant RBCC units of about 55 mL each. Blood transfusion services produce small volume units in order to reduce cost and wastage. Clinicians who expect premature neonates with low birth weights of less than 1500 g to be multiply transfused will place these patients on the neonatal limited donor exposure programme where units processed from one donor are reserved for a particular infant. This reduces the risk of infection and the production of allo-antibodies as patients will then only be exposed to one set of donor antigens.^6,17^

It is known that blood stored for long periods contain storage lesions that are not well tolerated in neonates requiring multiple transfusions.^18^ Hyperkalaemia and arrhythmia have been associated with ill infants receiving large volumes of blood. This may be due to high potassium levels present in stored RBC products, which may be caused by environmental heating or cellular degeneration caused by irradiation.^19^ Guidelines stipulate that blood used for an exchange or intrauterine transfusion for hyperkalaemic neonates to be less than 5 days old and infused within 24 h post-irradiation.^6,20,21^

### Irradiation

Gamma irradiation prevents the cells from engrafting and initiating an immune response against the host, as it inhibits the proliferation of T lymphocytes when lymphocyte DNA becomes damaged. Irradiation mainly affects RBC components and not granulocytes or platelets.^22^ As irradiation is the preferred method to prevent transfusion-associated graft versus host disease, most blood transfusion establishments use the freestanding ^137^C irradiator, where the cellular components are exposed to irradiation dosages between 25 Gy and 50 Gy. Blood may be irradiated 14 days post donation and stored for another two weeks without losing RBC viability.^6^ Clinical indications include cellular donations from blood relatives, donors who are homozygous for shared human leukocyte antigen haplotypes, neonatal intrauterine or exchange transfusions and allogeneic bone marrow transplant recipients.^23^ RBC membrane impairment due to irradiation causes an increase in supernatant potassium ions, plasma haemoglobin and LDH levels with a decrease in pH concentration, indicating that gamma irradiation exacerbates storage lesions.^24,25^

### Percentage plasma haemolysis

RBC haemolysis may be due to factors such as the storage period, the presence of leucocytes in unfiltered blood, mechanical injury during filtration, bacterial contamination during donation or component processing malpractices.^26,27^ Haemolysis increases the oxygen affinity of haemoglobin, which reduces oxygen transfer to the tissues. The biochemical reaction of plasma haemoglobin and nitric oxide may cause endothelial dysfunction, leading to intravascular thrombosis, leucocyte adhesion and possible vasoconstriction.^28^

South African blood transfusion establishments concur with the European Council ruling that stipulates that the haemolysis levels of stored RBC products should be less than 0.8% at the end of the storage period, whereas the United States Food and Drug Administration specifies less than 1%. Both councils insist on a 75% survival rate for transfused RBCs 24 h post-transfusion.^3^ Despite various methods available to measure haemolysis, no standardised method exists as each criterion may be measured in various ways. One study reported a coefficient of variation of approximately 55% between 14 laboratories measuring percentage of plasma haemolysis.^27^

The common practice of visually checking RBC products before they are issued from the blood bank is often incorrect, subjective and may result in gross overestimation. This may lead to these units being discarded, as dark pink supernatant discoloration may demonstrate plasma haemoglobin levels as low as 0.09% haemolysis.^29^

### Monitoring the quality of blood

A quality management system ensures that the quality of blood and blood components is continuously monitored to allow patients to receive the safest possible blood and to provide a service to stakeholders (donors, clinicians, patients, business partners and employees). Management ensures that facilities are adequately equipped and have trained or qualified staff on site to collect blood donations and subsequently process, test and issue blood and blood products. In South Africa, procedures are completed according to a departmental standard operating procedure document and staff must comply with the relevant Acts and the standards of practice for blood transfusion. Quality management systems also ensure that all equipment used for laboratory testing have been validated and routinely calibrated and that reagents utilised are quality controlled according to a prescribed method. Deviations from documented work procedures are recorded, investigated and appropriate corrective action taken. The quality indicators are continuously monitored and documented, such as during annual internal and external audits, staff proficiency tests, competency assessments and the monthly blood components quality control assessments.

The purpose of this study was to compare biochemical and haematological storage lesions in irradiated and non-irradiated RBC components that occur during the standard storage period to assess the effect of irradiation on WB and paediatric RBBC, both products are used in neonatal transfusion, and to determine whether current South African policies regarding the storage period of irradiated products need be modified.

## Methods

### Ethical considerations

Each donor completed a donor questionnaire prior to WB donation where they agreed that their donation may be used for research purposes. Available levels of blood stock were not compromised and donor confidentiality was maintained by using barcoded serial numbers. The Chief Executive Officer or Medical Director at the blood transfusion establishment provided a letter of consent for the research study to take place at the establishment’s headquarters in Cape Town, South Africa. Ethical approval from the Health and Wellness Sciences Research Ethics Committee from the Cape Peninsula University of Technology (study approval number: CPUT/HW-REC 2014/H10) was obtained. All ethical, scientific standards and good clinical practice were maintained throughout the study.

### Study population

Voluntary, non-remunerated donors residing in the Western Cape region of South Africa were selected according to the criteria for the protection of the donor and recipient as indicated in the standards of practice for blood transfusion in South Africa.^30^ Donors unable to fulfil the criteria and first-time donors were excluded from the study.

### Study design

This was a random sample research design, and the products tested included WB, buffy coat-poor RBCC, pre-storage leucocyte-reduced RBCC and paediatric RBCC. Eighty WB units were collected into collection bags containing citrate phosphate dextrose Terumo (quadruple pack with diversion pouch) on the same day. WB units were randomly selected for processing into the different products. Each test group consisted of 10 irradiated RBC units per product and the control group comprised 10 non-irradiated counterparts. A WB unit was separated into components via centrifugation using a Sorvall RC 12 BP Plus centrifuge (Thermo Fisher Scientific, Waltham, Massachusetts, United States) and centrifuged at 3140 rpm for 12 min at 4 °C to produce RBCC, fresh frozen plasma and cryoprecipitate products. A WB unit was also centrifuged at 3140 rpm for 10 min at 22 °C to produce RBCC, fresh frozen plasma and platelets. This was done to not compromise the stock levels. Plasma and buffy coat were extracted using a T-ACE II extractor (TerumoBCT, Lakewood, Colorado, United States). The units placed in the test group were gamma irradiated the day after processing using a Gammacell 3000 irradiator (Nordion Inc., Ottawa, Ontario, Canada). The irradiator was loaded with two components at a time with a minimal gamma exposure of 2574 cGy and a central dose of 2932 cGy (range: 25 Gy to 50 Gy). A machine-cycle printout was attached to each irradiated product.

### Sampling

A diversion pouch was aseptically attached to the RBC unit by using a TSCD^®^ II Sterile Tubing Welder (TerumoBCT, Lakewood, Colorado, United States). After the pouch was filled with the required amount of blood, the tubing was hermetically sealed using the tube sealer and the unit was returned to the fridge to be stored at 1 °C to 6 °C. Supernatant for testing was obtained by centrifuging the test sample at 3000 rpm for three min. This procedure was repeated on Days 7, 14, 21, 28, 35 and 42.

### *In vitro* measurements

Sample supernatant was used to test potassium, phosphate, sodium, glucose and LDH using the ABX Pentra 400 chemistry analyser (Horiba ABX SAS, Montpellier, Cedex, France). The pH was measured using a desktop pH meter (Amtech Laboratory Services, Cape Town, South Africa). The haematological levels for the full blood count, haemoglobin, haematocrit, mean cell volume, mean corpuscular haemoglobin and the mean corpuscular haemoglobin concentrate were measured using the ABX Pentra XL 80 (Horiba ABX SAS, Montpellier, Cedex, France). The Hemocue^®^ Plasma/Low Hb System (SE-262-71, Hemocue AB, Ängelholm, Sweden) was used to measure the plasma haemoglobin levels. The formula for calculating the percentage of plasma haemolysis is detailed below.

Equation 1: Calculation for percentage plasma haemolysis (Adapted from Leitner, 2001)^22^

$$\begin{array}{l} \text{Percentage plasma haemolysis}\\ =\frac{(100-\text{Haematocrit})\times \text{Plasma}(\text{supernatant})\text{haemoglobin}}{\text{Total haemoglobin}} \end{array}$$

Testing for the haematological factors, biochemical indices and routine donor testing commenced within the accepted 72 h post-collection and is known as Day 1.

### Statistical analysis

Data were documented using an Excel spreadsheet (Excel 2013, Microsoft Corporation, Redmond, Washington, United States). Statistical analyses of the test results were performed using Excel 2013, as well as Prism 5, 2007 (GraphPad Software, Inc. San Diego, California, United States). Statistical comparison of the non-irradiated (control group) and irradiated (test group) was completed using paired *t*-test for single time points and analysis of variance for multiple time points during the storage period. Multi-comparisons to define specific differences between the control and test groups at specific time points were performed using the post-hoc Bonferroni statistical calculations. Results are expressed as mean ± standard deviation; a *P*-value of less than 0.0 (*p* < 0.01) was considered significant.

## Results

The results for WB observed for the testing of biochemical analytes and haematology factors for the non-irradiated and irradiated units during the storage period are listed below, together with graphical illustrations.

### Biochemistry

**FIGURE 1:**
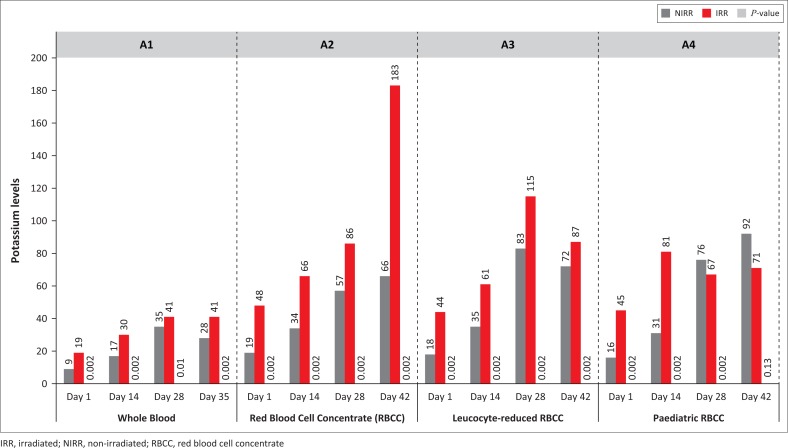
Potassium levels.

**FIGURE 2:**
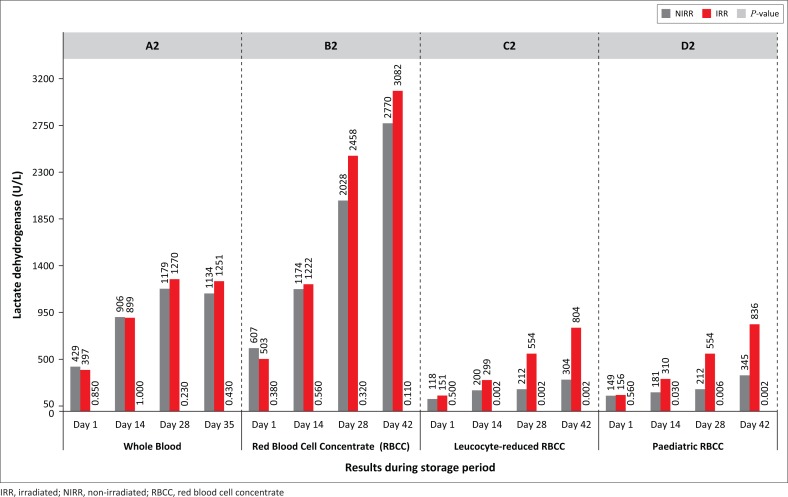
Lactate dehydrogenase (LDH) levels.

**FIGURE 3:**
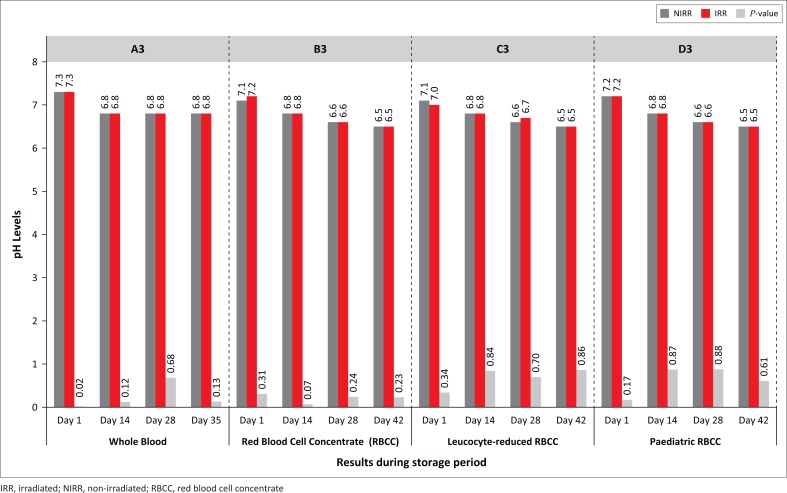
pH levels.

Statistically significant increases in serum potassium results were observed (Figure 1), except for WB Day 28 (*p* = 0.01) and paediatric RBCC on Day 42 (*p* = 0.13). Sodium levels decreased during storage, whereas the phosphate levels increased, except for the leucocyte-reduced non-irradiated RBCC and paediatric RBCC on Day 28 due to outliers. Most results were not statistically significant (*p* > 0.01), except for paediatric RBCC on Day 42 (*p* = 0.002). A reduction in serum glucose levels indicating glycolytic metabolism was observed for all non-irradiated and irradiated products, albeit with no statistical significance. LDH concentrations increased with statistical significance for leucocyte-reduced RBCC on Days 14, 28 and 42 (*p* = 0.002) and paediatric RBCC on Day 42 (*p* = 0.002) (Figure 2). LDH levels for non-irradiated WB increased by 164% and irradiated WB increased by 215%, whereas non-irradiated paediatric RBCC increased by 132% and irradiated units increased by 436%. A gradual decrease in pH levels was observed with no statistical significance (Figure 3).

### Haematology

**FIGURE 4:**
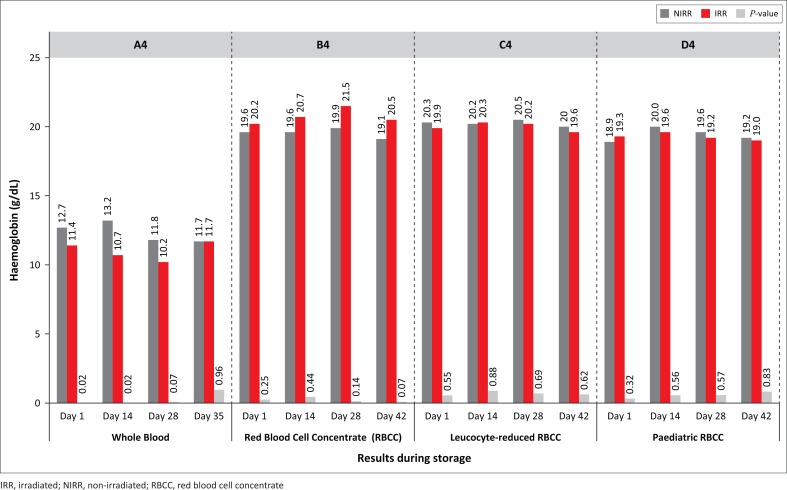
Haemoglobin (Hb) levels.

**FIGURE 5:**
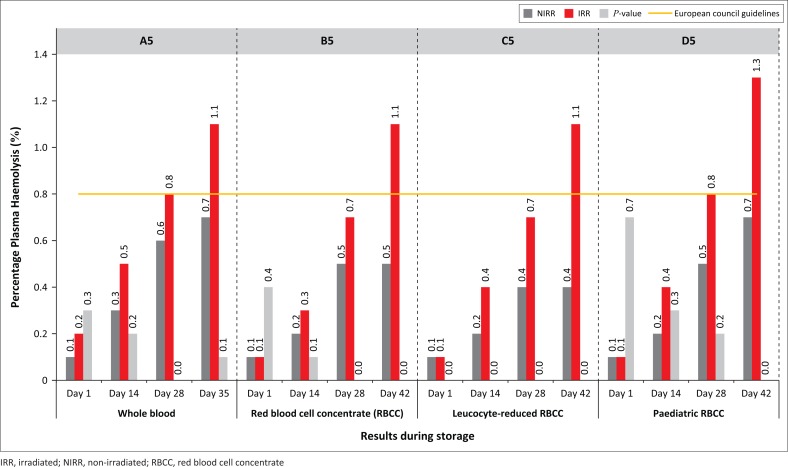
Percentage plasma haemolysis levels.

**FIGURE 6:**
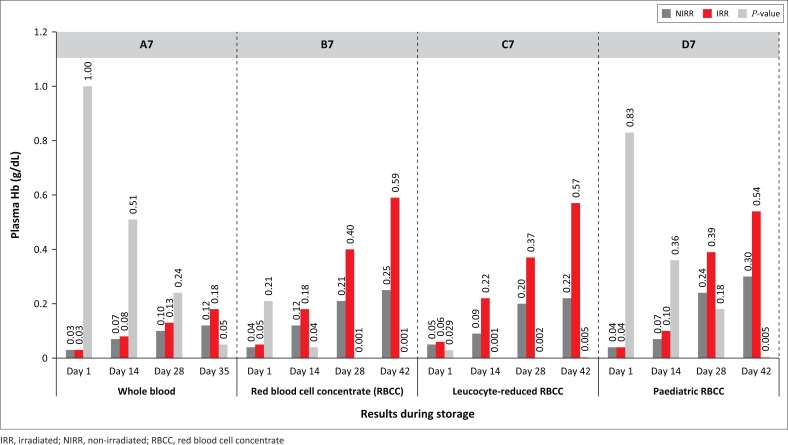
Plasma haemoglobin levels.

The haemoglobin levels for non-irradiated WB and RBCC decreased by 8% and 3% (*P*-values: 0.02–0.88) (Figure 4). The percentage of plasma haemolysis results indicate that by expiry all irradiated products exceeded the 0.8% haemolysis level as stipulated by the European Council, whereas non-irradiated levels were all below 0.8% (Figure 5). Statistically significant increases in plasma haemoglobin for RBCC were indicated on Days 28 and 42 (*p* = 0.001) and on Days 28 and 42 (*p* = 0.002) for leucocyte-reduced RBCC (Figure 6). Mean corpuscular volume results for irradiated WB decreased from 88.5 fl (Day 1) to 87.6 fl (Day 35). The haematocrit levels showed a gradual increase, whereas all products displayed *p*-values higher than 0.01 for both mean corpuscular volume and mean corpuscular haemoglobin levels during the storage period. Mean corpuscular haemoglobin concentrate levels displayed similar trends except for paediatric RBCC on Day 28 (*p* = 0.005), and *P*-values for erythrocyte levels ranged from 0.01 to 1.00. A reduction in leucocyte and thrombocyte levels for both non-irradiated and irradiated products was observed. Statistically significant values were observed for leucocyte-reduced RBCC on Day 1 (*p* = 0.006), Day 14 (*p* = 0.002) and Day 28 (*p* = 0.001).

## Discussion

This study aimed to determine whether there are any significant differences between non-irradiated and irradiated RBC products produced in Cape Town and to establish whether the South African policy regarding irradiated RBC products should be amended. While current literature indicates that gamma irradiation aggravates storage lesions in RBC products, less is known about irradiated leucocyte-reduced components.^31^ The blood transfusion establishments in South Africa do not comply with the universal leuco-reduction policy due to the cost.^6^

Although gamma irradiation exacerbates storage lesions, it is the accepted method to prevent transfusion-associated graft versus host disease.^24^ The irradiated RBC products in our study had higher percentage of haemolysis levels from Days 28 to 42, which has also been observed by earlier studies.^22,25,29,32,34,35,36^ Blood transfusion establishments should be aware that there are many factors contributing to RBC haemolysis. Some of these factors include analyte differences between donors, diverse pre- and post-transfusion abilities of various donors, possible genetic inconsistencies, pre-analytical factors and component processing strategies.^3,26,34^ All used units were sent to the microbiology laboratory to be examined for bacterial contamination. The results were negative.

The results for non-irradiated paediatric RBCC indicate statistically significant potassium results on Day 28. While top-up neonatal transfusions are mostly small volume (10–20 ml/kg), it is suggested that blood be used before Day 28 even though the risk to hyperkalaemic neonates is minimal due to the small plasma volume. However, for large volume transfusions such as neonatal exchange transfusions or in acute blood loss, WB less than 5 days is used to prevent hyperkalaemia and low 2, 3-diphosphoglycerate levels.^6^

Our results confirm most previous outcomes regarding filtered RBCC that have been irradiated not later than Day 14.^32,33^ It also proves that leucocyte-reduction of RBC products moderate haemolysis as a decrease in haemolysis was observed when non-irradiated Day 42 RBCC was compared to non-irradiated, pre-storage, leucocyte-reduced Day 42 RBCC. Our results also indicate that cellular degeneration is lower when leucocyte-reduced non-irradiated RBC products are compared to their irradiated counterparts and confirms that RBC leucocyte-reduced products improve blood safety and efficacy.^34^

### Limitations

Donor blood was not tested prior to component separation and subsequent irradiation; thus, there were no baseline measurements. RBCC were produced by centrifuging WB at two different centrifugation temperatures, instead of a single temperature of 4 °C. An improved strategy would be to use one RBC product and divide it into two equal aliquots where one bag is irradiated and the second bag used as the control. Additionally, it was an oversight not to include the irradiation of blood on Day 14 and keep it stored until expiry (per United States guidelines).

### Recommendations

Despite controversial debates regarding the transfusion of RBCC products, it still remains a popular treatment resource. The published results of meta-analysis regarding storage lesions should be carefully reviewed before policies relating to transfusion medicine are amended. The scientific community should be contemplating improvements to storage strategies, such as anaerobic storage, nutritive additives, proteomic-based biomarkers and an alternative to gamma irradiation for the prevention of graft versus host disease. Oxidative stress may occur, and therefore the addition of antioxidants to units of blood could possibly decrease lipid peroxidation and prevent leakage of potassium, LDH and haemoglobin into the plasma.

### Conclusion

The outcome of this study confirms that gamma irradiation exacerbates RBC storage lesions during storage at 1 °C to 6 °C for up to 42 days. Increases in potassium, LDH and haemolysis levels were observed, which may result in serious implications when transfused to immunocompromised patients. This study further observed significant differences between irradiated WB and paediatric RBCC due to differences in plasma volume. Significant differences were also demonstrated between adult irradiated, pre-storage, leucocyte-reduced RBCC and paediatric RBCC for glucose, pH levels, plasma haemoglobin levels and plasma haemolysis. Although these products are both leucocyte-reduced, the irradiated paediatric RBCC undergoes further product modification. The products tested yielded similar results to other studies in developed countries that leucocyte-reduction improves blood safety and efficacy. While South African blood transfusion services do not comply with the universal leuco-reduction policy, it is recommended that for now, the South African transfusion medicine policy remain unchanged, as it is not cost-effective to only produce leucocyte-reduced products due to the country’s current health issues.

## Reliability

The evaluations of the testing performed in this study were completed using equipment, methods and corresponding reagents according to the manufacturers’ instructions and relevant standard operating procedures. Blood and samples were collected from the donor population in the Western Cape region of South Africa. The equipment and analysers with readily available corresponding reagents are well known in the field of immunohaematology and therefore the methodology used in this study can be applied elsewhere in the world.
